# A DM-ELM based classifier for EEG brain signal classification for epileptic seizure detection

**DOI:** 10.1080/19420889.2022.2153648

**Published:** 2022-12-15

**Authors:** Shruti Mishra, Sandeep Kumar Satapathy, Sachi Nandan Mohanty, Chinmaya Ranjan Pattnaik

**Affiliations:** a Department of Computer Science & Engineering, Vellore Institute of Technology, Chennai, india; b School of Computer Science &Engineering, VIT-AP University, Amaravati, India; c Department of Computer Science & Engineering, Ajay Binaya Institute of Technology, Cuttack, India

**Keywords:** Electroencephalogram (EEG) signals, discrete wavelet transform (DWT), extreme learning machines (ELM), moth flame optimization (MFO)

## Abstract

Epilepsy is one of the dreaded conditions that had taken billions of people under its cloud worldwide. Detecting the seizure at the correct time in an individual is something that medical practitioners focus in order to help people save their lives. Analysis of the Electroencephalogram (EEG) signal from the scalp area of the human brain can help in detecting the seizure beforehand. This paper presents a novel classification technique to classify EEG brain signals for epilepsy identification based on Discrete Wavelet Transform and Moth Flame Optimization-based Extreme Learning Machine (DM-ELM). ELM is a very popular machine learning method based on Neural Networks (NN) where the model is trained rigorously to get the minimized error rate and maximized accuracy. Here we have used several experimental evaluations to compare the performance of basic ELM and DM-ELM and it has been experimentally proved that DM-ELM outperforms basic ELM but with few time constraints.

## 1. Introduction

Epileptic seizure is one of the lethal neurological [1,2] disorder. It has taken up approximately 50 million people throughout the world. There has been technically many treatments including the advanced treatments for dealing with the epileptic seizure detection. But despite of this fact, there exists many individuals who are skeptical to the drug and are characterized to the obstinate seizures. Study has often perceived that, if the patient is diagnosed in the right time, the chances of survival without any seizure increase a lot. The main challenge in this entire study lies with the signal variations. Many professional studies suggested, that the signal spectrum [3,4] study could lead to many significant information about human brain but there is mostly variation that exists in the signal peaks and frequency. This makes it difficult in diagnosing the presence of seizure. Currently, several ongoing researches based on the brain classification [5–7] of EEG Signal is actively on to detect the anomaly, ambiguity, epileptic seizure, sleep disorder, etc. As we are aware of this fact, that human brain is built primarily with millions and trillions of neurons that actively play an most role in controlling the activities and behaviors in the human body by instantiating and stimulating the neurons. They deal with these active neurons, regulate them, and contribute in stimulating the other active neurons around them. These activated neurons act as an information carrying agents between the human brain and body. They also act as a synchronizing medium for balancing the human behaviors based on the environmental condition. These EEG signals [8] are acquired from the brain scalp area and are categorized to different types based on their frequency distribution. Analysis of this signal plays a major role in detecting the epileptic seizure.

These seizures need to be detected and well classified earlier to avoid deaths and unwanted medical issues. For the classification of the signals, ELMs [9] can be used alongside the discrete wavelet transform. ELMs being a modified version of neural network algorithm is quite fast in calculation and non-linear processing ability. In many ways, this technique has been proved beneficial because of its fast learning capacity and good linear processing capabilities. Due to this ELM has the potential for detecting non-linearity and seizure attacks in the EEG signal that has varying frequency. Similarly, Discrete Wavelet Transform (DWT) [10] has been quite an effective technique in the signal decomposition mostly used of non-stationary signals. The technique is quite renowned and well versed in extracting the features for building classifier model. Our study primarily focusses on developing a classifier that would be an amalgamation of the DWT and ELM technique for the purpose of EEG signal classification.

The paper is segregated into different sections: Section 1 provides us an insight about the EEG classification, epileptic seizure and role of the techniques in brief; section 2 provides us a background idea/work related to EEG signal analysis and epileptic seizure detection. Similarly, section 3 details out the methodologies used in our study followed by the section 4, which presents our proposed model. Section 5 provides an insight about the result analysis part and ultimately, we conclude in out last section while also stating about the future scope.

## 2. Literature survey

This section presents us with some well-versed work by different researchers in the area of EEG signal analysis, DWT and ELMs. These includes:

Jing et al. [11] proposed a method for classification and identification of the seizure/epileptic seizure from the EEG signals by using the signal enhancement technique. This technique focussed on sliding the weighing window for removing the noise and transforming the target signal. Anuragi et al. [12] used phase-space representation for epileptic seizure classification in EEG signal by using ensemble learning mechanism. They decomposed the signals into sub-bands using empirical wavelet transform method that is based on Fourier Bessel series expansion. Soilaja et al. [13], proposed a decomposition method from scalp area EEG signal that extracts curve length features using decision tree classifier. Another method for seizure detection was proposed by Mehala et al [14], where they used SVM classifier for LP norms feature detection using Fourier Intrinsic method. Hussain et al. [15] used L1 penalized regression method for seizure detection. They used L1 penalized regression for feature vector finding and using random forest as an input factor to classifier. Radman et al. [16], thought of a fusion-based technique for feature extraction leading to seizure detection. They used time and frequency as feature vector and applied Demster-Shafer evidence theory for improvised feature extraction.

Nandini et al. [17] used dropout technique for classification based on ELM technique where it was proved that the training time was far lesser than the existing unmodified ELM technique. Barua et al. [18] proposed an automated Parkinson’s detection system by using the aspirin pattern that exists in EEG signal. They used multi-level feature model for generating new pattern, statistical moments and absolute pooling. Sankar et al. [19] proposed a higher order statistical technique for disease detection using EUS ensemble method that used tree classifier. Another technique proposed by Anjum et al. [20] uses any fold cross-validation for disease detection using EEG signal. Similarly, Zu et al. [21] proposed a deep recurrent neural network technique for seizure detection in EEG signal. They focussed more on the issues of sensitivity and specificity.

Similarly, Yuvaraj et al. [22] proposed a higher order spectrum feature-based approach for disease detection through EEG signal with many fold cross validation as a metric. Oho et al. [23] also proposed a technique using convolutional neural network method with 13 layers for disease detection. Another work based on dimensionality reduction for disease detection was proposed by Gunduz et al. [24]. Similarly, Naghas et al. [25] also proposed a combination of magnetoencephalography and EEG signals for disease detection.

## 3. Materials and methods

This research work involves mainly three different phases. In the first phase, the data pre-processing is done for converting EEG raw signal data set into a proper sample-feature format data set. In this phase, we have used Discrete Wavelet Transform (DWT) for decomposition of signals into various levels. Then, from each level the detailed coefficients are taken except in the last level from where both detailed and approximation coefficients are taken into consideration. After signal decomposition the various statistical features have been calculated for different coefficients using which the final data set has been constructed. The next phase consists of a classification process that has been carried out in two phases. In one phase, the optimal parameters for classifier has been calculated using Moth-Flame Optimizer (MFO). And, in the third and final phase classification has been done using Extreme Learning Machine (ELM).

### 3.1. Dataset description

In this work we have collected the data from publicly available resources for EEG data [xx]. This dataset is publicly available by University of Bonn by department of Epileptology. There are total five datasets available A to E each with 100 single channel EEG of 23.6 sec duration. These five datasets are combined in different ways to form three different sets, SET1, SET2 and SET3. SET1 is a combination of A, D and E. SET2 is a combination of B, D and E. SET3 is a combination of C, D and E.

### 3.2. Methodologies used

#### 3.2.1. Discrete Wavelet Transform (DWT)

**Figure 1.**
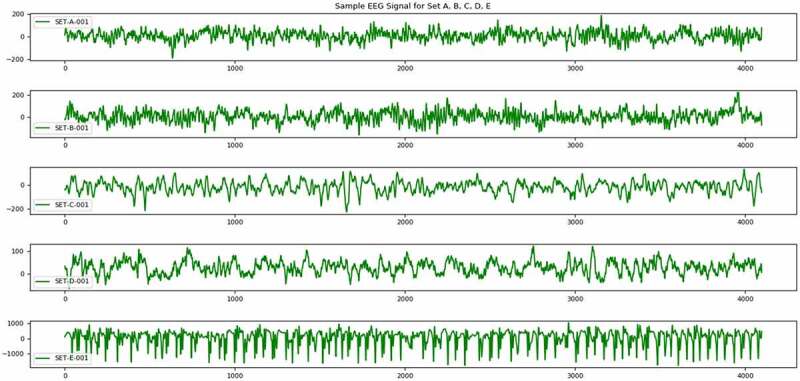
Sample EEG signal from each set A, B, C, D and E.

**Figure 2.**
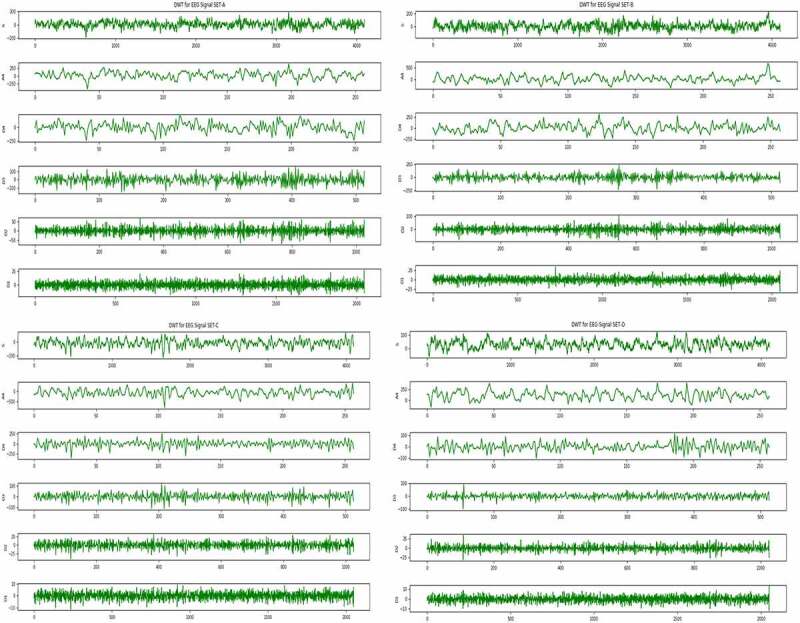
Sample EEG signal from each set A, B, C and D after decomposition using DWT.

**Figure 3.**
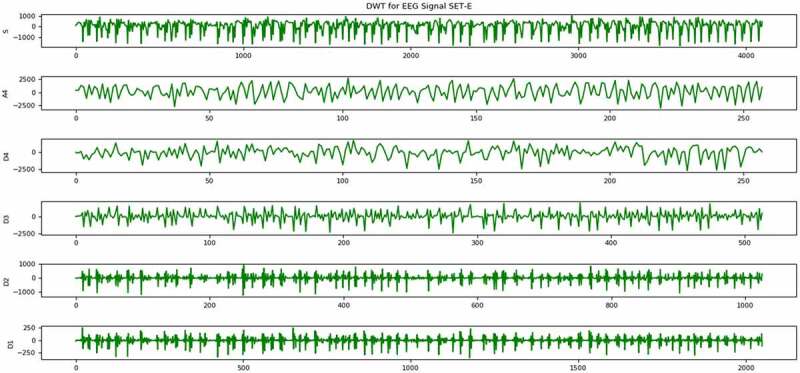
Sample EEG signal from set E after decomposition using DWT.

DWT is a very powerful and effective signal decomposition technique, which is generally used for analysis of transient or non-stationary signals [26]. EEG signal is one such type of transient signal, as its frequency is not constant with respect to time. These types of signals are very difficult to analyze. Proper analysis of these type of signals can help in identification of several abnormal patterns in the signals. One of the abnormalities that we are trying to find out in this work is epilepsy. As the signals are in raw format, which cannot be utilized for any kind of data mining task, hence DWT is applied for decomposition of signals and after those statistical features have been extracted to construct a proper sample feature format data set to carry out the data mining task (Figure 1 and 2). The core part of this technique is the mother wavelet function, which is used in this work is Daubechies wavelet of order 2 and decomposed up to level 4 (Figure 3).

#### 3.2.2. Moth-Flame Optimization (MFO)

Optimization algorithms have their own charm in finding the best possible solutions for any kind of specific problem. The complexity of the problems gradually has increased with each passing time. Hence, the need for advancement in the optimization technique is more compared to earlier. The MFO algorithm [27] has been inspired from the family of Moths, which are typically a varied insect similar to butterflies. Being a nature-inspired algorithm, they mostly navigate and move around at night to maintain a fixed angle position to the moon. Mostly they create a spiral scrolling path called as transverse orientation for navigation. The moths mostly get confused and tricked with the human made artificial lights and think it as moon light. So, they try to maintain this orientation and angle with those artificial light too. This at times because very dangerous and deadly due to its spiral angle near to the light. The MFO algorithm deals with two important aspects: candidate solutions i.e. the moths and the problem’s variables that are indicative of the moths’ positions. The moths have the capabilities to fly in many dimensional spaces like 1-D, 2-D, 3-D or hyper-dimensional space with changing positional vectors. Moths usually fly around the flames hence; the flames and the moths are taken to be the solutions but the problem only lies with the update and the treatment at each iteration. The mathematical formulation of the flying behavior of moths according to the flames can be described as below:

(1) $$T_{i}=H\left(T_{i},M_{j}\right)$$

Where $T_{i}$ represents i^th^ moth and $M_{j}$ represents j^th^ flame. H is the helical function satisfying few predefined conditions. According the conditions the helical function can be mathematically formulated as below:

(2) $$H\left(T_{i},M_{j}\right)=L_{i}*e^{\;\beta \alpha }*cos\left(2\pi \alpha \right)+M_{j}$$

Where $L_{i}$ is the linear distance from i^th^ moth and j^th^ flame, β is the logarithmic helix shape constant predefined, α is the path coefficient between −1 to +1.

#### 3.2.3. Extreme Learning Machines (ELM)

One of the most popular algorithms is ELM based on the feed-forward neural network algorithm that is used to improve the efficiency and speed of the network. These algorithms are typically used for classification for its outstanding performance in the real time learning tasks. The algorithm converges faster than any traditional computational algorithm and provides promising results throughout. The algorithm is typically used in many learning processes that includes the sequential learning, batch learning, etc., for the speed and accuracy toward the result generation. One of the greatest benefits of this algorithm is the ability to train itself faster than any other algorithm. Also, they do not use the sluggish gradient descent-based learning algorithms rather are based upon the Single-Hidden Layer Feed Forward Neural Networks. Even though frequently used for classification, they can also be used for regression, clustering, sparse approximation, compression and feature learning. The ELM model performance is so promising that at times they outperform Support Vector Machines (SVM) depending on the application for which it is used. The detailed mathematical description of ELM is given in equation 3 with all the symbols annotated.

(3) $$f_{H}\left(x\right)=\overset{H}{\underset{i=1}{\sum }}\overset{N}{\underset{j=1}{\sum }}v_{i}h\left(w_{i}*x_{j}+\beta_{i}\right)$$

Where H: number of hidden units, N: Number of training input samples, v: weight vector between hidden layer and output layer, w: weight vector between input and hidden layer, h: activation function for ELM, x: input vector: bias vector.

### 3.3. Experimental Design of DMELM: A Hybridized ELM Model

**Figure 4.**
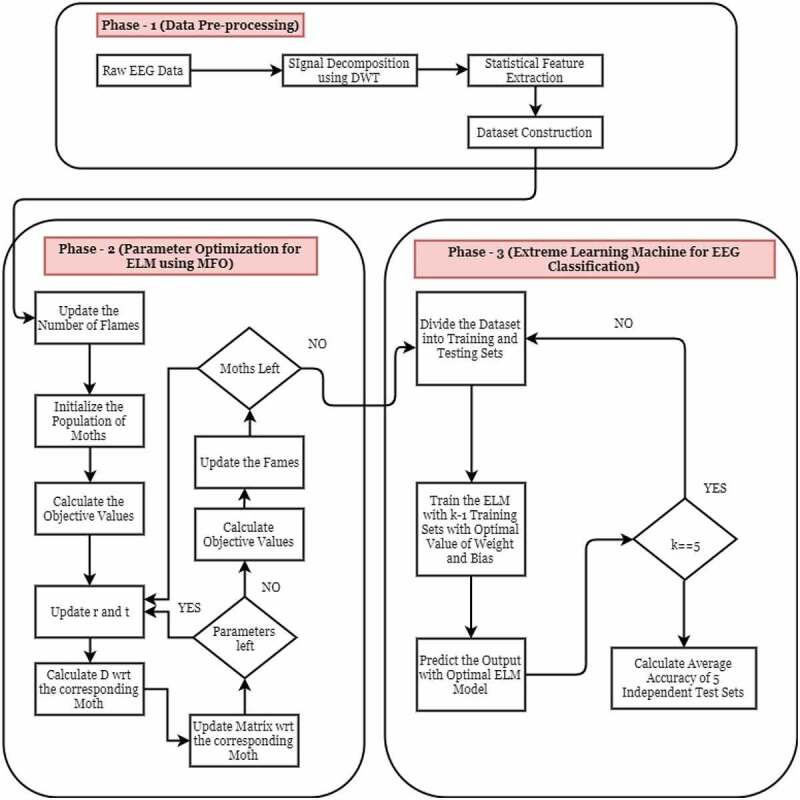
Architecture of proposed model (DMELM).

Above Figure 4 shows the proposed experimental design architecture for DM-ELM classifier for EEG signal classification for epileptic seizure detection. As shown in figure there are basically three phases through which the whole process is implemented. In the first phase data collection and pre-processing have been done. The raw EEG data collected are not in the form of a proper dataset on which machine learning algorithms can be applied. Hence feature extraction has been done using DWT signal decomposition technique. From these decomposed signals various statistical measures have been used to construct the final dataset. In the second phase the parameters of ELM responsible for accurate classification have been optimized using MFO algorithm. Finally in the third phase ELM classifier have been developed with optimized parameters for classification of EEG dataset for epileptic seizure identification and different measures are used to measure the performance of classifier.

## 4. Results and Discussion

**Figure 5.**
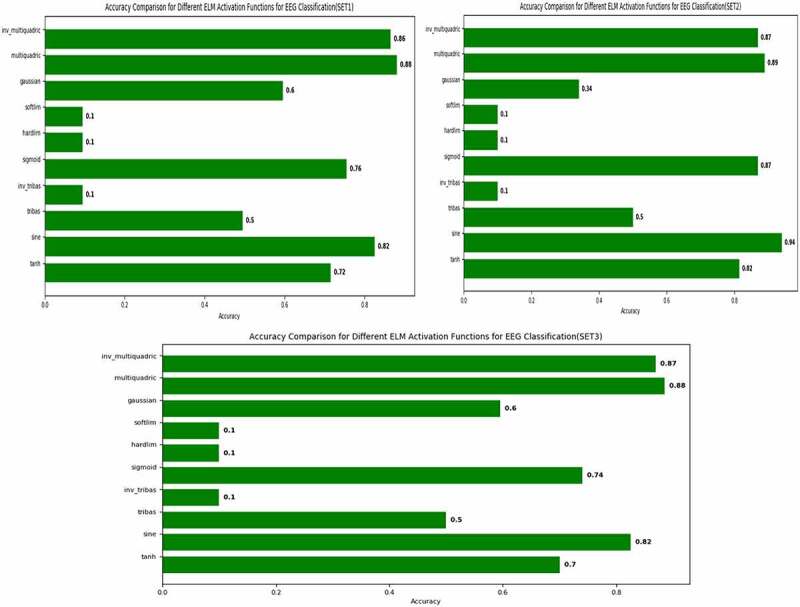
Accuracy comparison of different Afs for SET 1, 2, and 3 using ELM.

All the experimental evaluations for this work have been carried out using Python 3.7 with the support of different libraries. All the experiments carried out using core i7 processor with 3.4 GHz speed and 8GB RAM with GPU support. ELM classifier has been tested with several activation functions such as sine, tanh, tribas, inv_tribas, sigmoid, hardlim, softlim, gaussian, multiquadric and inv_multiquadric etc. Figure 5 shows three graphs containing all the information regarding comparison of different activation functions for three different EEG SETs constructed earlier using proposed DM-ELM.

Table 1 shows the parameter setups used for different techniques used to carry out experiments for analysis of EEG signal in epilepsy identification.

**Table 1.** Parameter setup for different techniques used.

Techniques used	Parameter	Value
Moth Flame Optimization	Max_Iteration	1000
	Lower Bound (lb)	100
	Upper Bound (ub)	100
	Number of search agents	50
	Dimension of search space	30
	Objective function	$f_{H}\left(x\right)=\overset{H}{\underset{i=1}{\sum }}\overset{N}{\underset{j=1}{\sum }}v_{i}h\left(w_{i}*x_{j}+\beta_{i}\right)$
Extreme Learning Machine	Activation function	Inverse Multiquadric
	Number of hidden layers	500

Figure 5 shows the performance comparison of different activation functions for EEG signal in three different sets. This experiment was carried out just to understand the behavior of different activation functions of ELM and how it can be best used for our domain of EEG signal classification. Table 2 shows the overall accuracy of different activation functions for set 1, 2 and 3. From this table and above figures it can be concluded that the performance of ELM with optimized parameters of multiquadric activation function gives highest accuracy compared to all others with only one deviation that is for SET 2 activation function sine gives more accuracy compared to multiquadric function. Hence now onwards for all implementations multiquadric activation function was taken to carry out all experiments.

**Table 2.** Accuracy comparison for different activation functions for SET 1, 2 and 3 using ELM.

ELM Activation Functions	Dataset		
	SET-1	SET-2	SET-3
tanh	0.71 (± 0.11)	0.82 (± 0.17)	0.70 (± 0.09)
sine	0.82 (± 0.10)	0.94 (± 0.06)	0.82 (± 0.09)
tribas	0.49 (± 0.45)	0.50 (± 0.45)	0.50 (± 0.45)
inv_tribas	0.11 (± 0.21)	0.10 (± 0.20)	0.10 (± 0.20)
sigmoid	0.76 (± 0.10)	0.87 (± 0.11)	0.74 (± 0.10)
hardlim	0.11 (± 0.21)	0.10 (± 0.20)	0.10 (± 0.20)
softlim	0.11 (± 0.21)	0.10 (± 0.20)	0.10 (± 0.20)
gaussian	0.59 (± 0.22)	0.34 (± 0.32)	0.59 (± 0.21)
multiquadric	0.88 (± 0.07)	0.89 (± 0.13)	0.89 (± 0.06)
inv_multiquadric	0.86 (± 0.11)	0.87 (± 0.12)	0.87 (± 0.10)

**Figure 6.**
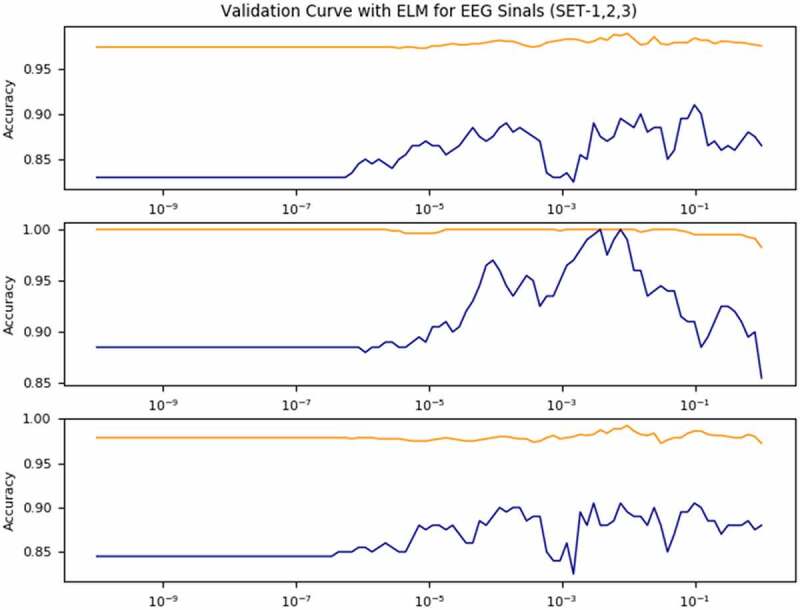
Validation curve for EEG signal classification using DM- Extreme Learning Machine (ELM) with three different categories of dataset (Set1, Set2 and Set3).

Figure 6 shows comparison of validation curve for DM-ELM and ELM classifier using multiquadric activation function for three different EEG datasets in epileptic seizure identification.

For understanding the performance of DM-ELM in a more comparative way, there are few benchmark machine learning algorithms that have been used and the same task has been performed. As shown in Tables 3, 4 and 5 Multilayer Perceptron Neural Network, Naïve Bayesian and regular ELM has been used for this comparison. From these comparisons it can be concluded that DM-ELM outperforms compared to all other techniques in EEG signal classification for detection of epilepsy for all three categories of datasets.

**Table 3.** Performance comparison for different machine learning techniques for SET1.

Classification techniques	Accuracy	Specificity	Sensitivity	F-score
MLPNN	0.84 ± 0.17	0.81 ± 0.12	0.82 ± 0.11	0.82 ± 0.10
Naïve Bayesian	0.78 ± 0.13	0.79 ± 0.15	0.76 ± 0.03	0.78 ± 0.15
ELM	0.88 ± 0.07	0.85 ± 0.05	0.84 ± 0.06	0.82 ± 0.08
DM-ELM	0.92 ± 0.02	0.93 ± 0.12	0.91 ± 0.03	0.94 ± 0.13

**Table 4.** Performance comparison for different machine learning techniques for SET2.

Classification techniques	Accuracy	Specificity	Sensitivity	F-score
MLPNN	0.85 ± 0.11	0.84 ± 0.01	0.84 ± 0.14	0.85 ± 0.13
Naïve Bayesian	0.79 ± 0.09	0.78 ± 0.12	0.77 ± 0.07	0.77 ± 0.07
ELM	0.89 ± 0.13	0.88 ± 0.14	0.87 ± 0.12	0.90 ± 0.04
DM-ELM	0.95 ± 0.03	0.94 ± 0.13	0.96 ± 0.02	0.96 ± 0.13

**Table 5.** Performance comparison for different machine learning techniques for SET3.

Classification techniques	Accuracy	Specificity	Sensitivity	F-score
MLPNN	0.84 ± 0.14	0.82 ± 0.11	0.83 ± 0.17	0.84 ± 0.04
Naïve Bayesian	0.80 ± 0.12	0.79 ± 0.14	0.78 ± 0.13	0.79 ± 0.16
ELM	0.89 ± 0.06	0.88 ± 0.05	0.87 ± 0.03	0.88 ± 0.08
DM-ELM	0.96 ± 0.12	0.95 ± 0.13	0.97 ± 0.02	0.94 ± 0.03

## 5. Conclusion

EEG signal analysis for disease identification is a real challenge in medical domain that needs to be addressed through Artificial Intelligence techniques. Epilepsy is one of the severe brain disease that is very difficult to identify. Hence in this work we have proposed an optimized machine learning algorithm for automatic diagnosis of epileptic seizures by analyzing EEG signals recorded. Here we have used Extreme Learning Machine with multiquadric activation function optimized with Moth Flame Optimization algorithm and dataset constructed through Discrete Wavelet Transformation.
